# Detection of genetic variation in sandalwood using various DNA markers

**DOI:** 10.1007/s13205-016-0391-0

**Published:** 2016-02-13

**Authors:** Dimple M. Patel, Ranbir S. Fougat, Amar A. Sakure, Sushil Kumar, Mukesh Kumar, Jigar G. Mistry

**Affiliations:** Department of Agriculture Biotechnology, Anand Agricultural University, Anand, 388 110 India

**Keywords:** Diversity, ISSR, RAPD, Sandalwood, *Santalum*, SSR

## Abstract

In the present study, 20 sandalwood (*Santalum album L.*) genotypes were characterized using RAPD, ISSR and SSR markers. Twenty-five RAPD and twenty-one ISSR primers that generated clear and reproducible banding patterns amplified 225 and 208 bands, respectively, among 20 sandalwood genotypes. Out of 225, 181 (83.13 %) RAPD bands were polymorphic while out of 208, 156 (75.77 %) ISSR bands were polymorphic. The average polymorphism information content (PIC) for RAPD and ISSR was 0.84 and 0.86, respectively. A good correlation (0.96) was observed between the matrices produced by RAPD and ISSR primers. Though, there was high similarity among genotypes (0.79 for RAPD and 0.70 for ISSR), the observed genetic diversity was found good enough for the characterization of sandalwood genotypes. Cross-species transferability SSR markers developed in *S. austrocaledonicum* and *S. insulare* were found to be monomorphic. The results of the present investigation would provide valid guidelines for collection, conservation and characterization of sandalwood genetic resources.

## Introduction

Sandalwood (*Santalum album* L; 2*n* = 20) is one of the important tropical trees which is commercially known for its fragrance (Shashidhara et al. 2003). It is member of family *Santalaceae* and being as perfumery material it is commonly known as chandan. It is a small- to medium-sized hemiparasitic tree, distributed rather widely in India. Sandalwood is mostly confined to the South Indian states, especially Karnataka, Tamil Nadu and Kerala, and is indigenous to Peninsular India (Srinivasan et al. 1992).

*S. album* or Indian sandalwood is of great commercial value due to its fragrant heartwood which yields unique oil preferred for perfumeries, cosmetics, medicines and also in incense sticks industries. Sandalwood oil has antipyretic, antiseptic, antiscabietic, and diuretic properties and is also effective in the treatment of bronchitis, cystitis, dysuria, and diseases of the urinary tract. The seeds are used as diuretic, hypotensive, antitumorigenic, antiviral agents, and for treating a number of skin diseases (Kirthikar and Basu 1987; Desai and Shankaranarayana 1990).

Globally, with high economic value of sandalwood and its oil, sandalwood wealth in forests are declining due to overharvesting and illegal poaching in natural habitats (Naseer et al. 2012). This alarming genetic erosion condition indicates that there is need to conserve this commercially important tree species. To protect the species, efforts have been made to establish ex situ conservation gardens for sandalwood in India (Rao et al. 2011). But the conservation efforts and planning suffer from lack of information on the level and structure of natural genetic variability of sandalwood populations (Rao 2004). Hence, to examine the existing genetic variability, there is urgent need for systematic variability study in sandalwood.

Initially, isozymes served as reliable markers for genetic analysis in sandalwood (Rao et al. 1998, 2007a; Angadi et al. 2003) but this biochemical based marker was relatively low in abundance hence revealed low polymorphism. Moreover, like phenotypic markers, isozymes may also be affected by environmental conditions depending on the type of tissue used for the analysis. On the contrary, PCR-based molecular/DNA markers like RAPD, ISSR and SSR are dispersed throughout the genomes, more polymorphic due to its abundance, environmentally independent and are easier to analyse. A number of studies have been conducted to understand the genetic diversity of sandal using random amplification of polymorphic DNA (RAPD) (Shashidhara et al. 2003; Suma and Balasundaran 2004; Azeez et al. 2009), simple sequence repeat markers (SSRs) (Mohammed et al. 2012) and restriction fragment length polymorphism (RFLP) (Byrne et al. 2003). Due to unavailability of native SSR, most of the diversity analysis studies in sandalwood were conducted independently with RAPD, ISSR and cross-species transferable SSR markers. However, none has tried to assess the comparative accuracy and reproducibility of different markers for the characterization of sandalwood for better depiction of genetic diversity of sandalwood. The present investigation was initiated with the objective to assess and compare the efficiency of RAPD, ISSR and SSR markers in assessment of genetic diversity prevalent in Indian sandalwood collection.

## Materials and methods

### Plant material and DNA extraction

**Fig. 1:**
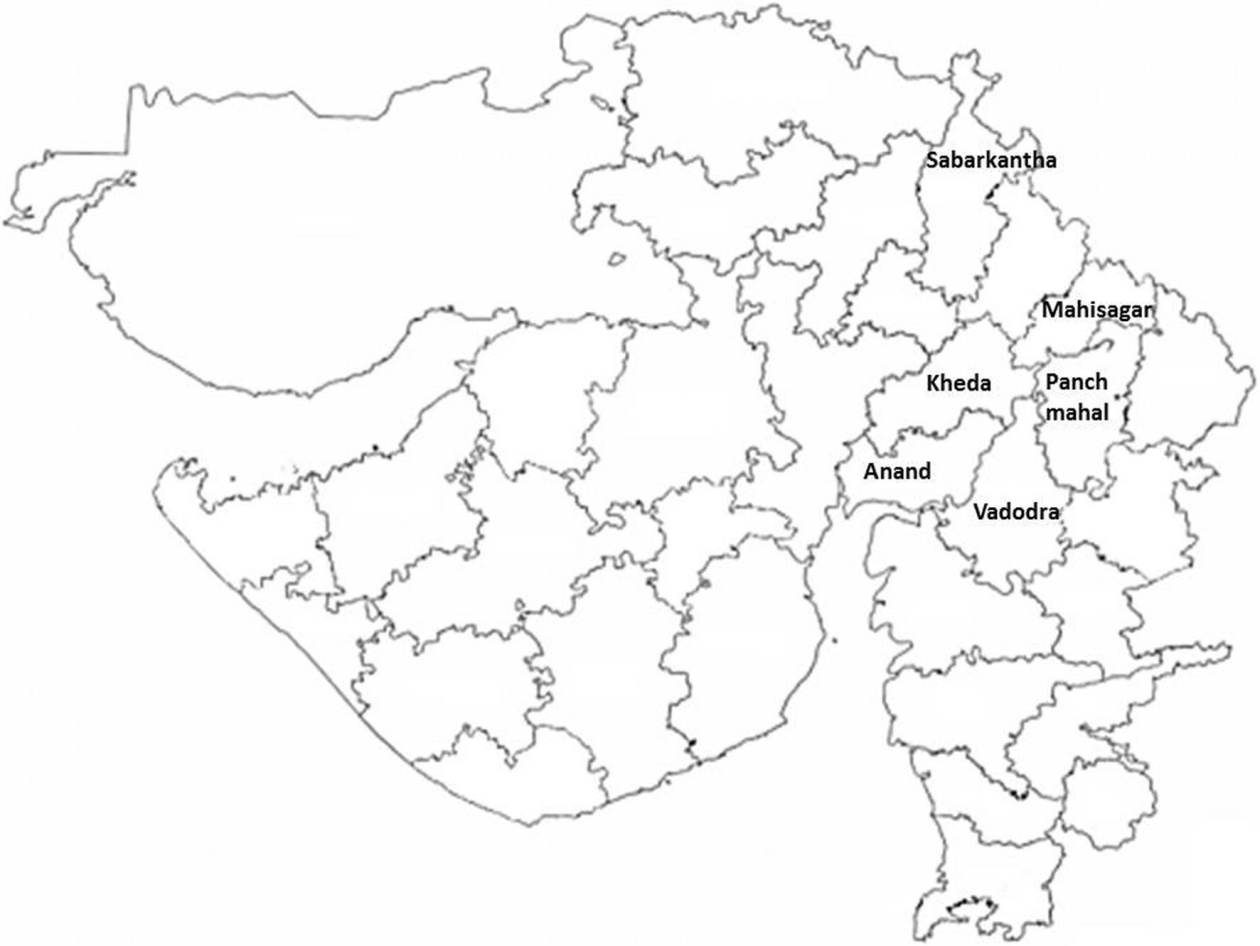
Geographical location of area in Gujarat state (India) selected for collection of sandalwood genotypes. (Map is only representative and distances are not scaled)

Leaf samples of a total of 20 sandalwood (Table 1) trees were collected from different places of Gujarat (Fig. 1). DNA from leaves was isolated using CTAB technique (Doyle and Doyle 1990), purified, and quantified using Nanodrop (Thermo scientific, USA). Finally, DNA was diluted to 20 ng/μl with TE buffer for PCR amplification.

**Table 1:** List of  sandalwood genotypes used in study with their place of collection

Accession	Place of collection	Latitude (N)	Longitude (E)
GSA-1	Horticulture farm, AAU, Anand	22.560869	72.954773
GSA-2	Jagnath Temple, Anand	22.560869	72.954773
GSA-3	NDDB campus, Anand	22.560869	72.954773
GSA-4	Kothamba, Mahisagar	23.016667	73.516667
GSA-5	Palla, Mahisagar	23.5223474	73.5741357
GSA-6	Laloda, Idar, Sabarkantha	23.8219944	73.0146996
GSA-7	Laloda, Idar, Sabarkantha	23.8219944	73.0146996
GSA-8	Halol, Panchmahal	22.2780157	73.7173256
GSA-9	Godhra, Panchmahal	22.76515	73.609383
GSA-10	Thasra, Kheda	22.7977535	73.2160825
GSA-11	Virpur, Mahisagar	22.2050438	71.0794901
GSA-12	M.S. University, Vadodra	22.3073095	73.1810976
GSA-13	M.S. University, Vadodra	22.3073095	73.1810976
GSA-14	Balasinor, Mahisagar	22.955891	73.336499
GSA-15	Sayaji Garden, Vadodra	22.3261207	73.2421344
GSA-16	Sayaji Garden, Vadodra	22.3261207	73.2421344
GSA-17	Sevaliya, Kheda	22.8100749	73.3443425
GSA-18	Umareth, Anand	22.695414	73.115857
GSA-19	Kharol, Mahisagar	23.0171961	73.471054
GSA-20	Tarapur, Anand	22.4888038	72.6579865

### PCR parameters and gel analysis

A total of 57 primers (25 RAPD, 21 ISSR and 11 SSR) were used for PCR amplification. PCR amplification was carried out in Biometra thermalcyclers (Germany). For PCR amplification, 25 µl reaction volume containing 2.5 µl template DNA (50 ng), 1× Dream Taq PCR buffer with MgCl_2_ (Fermentas, USA), 0.4 µl (5 U/µl) Taq polymerase (Fermentas, USA), 0.5 µl (2.5 mM each) dNTPs (Fermentas, USA) and 1 µl (10 pmol/µL) primer (MWG biotech, Germany) was used. RAPD amplification was performed according to Shashidhara et al. (2003) using decamer primers (Operon technologies Inc, USA; SIGMA-D, USA). RAPD-PCR was performed at an initial denaturation at 94 °C for 5 min, 38 cycles of 94 °C for 1 min, 38 °C for 1 min, 72 °C for 1.2 min, and final extension at 72 °C for 5 min. The optimal annealing temperature for ISSR primers was found to vary according to the base composition of the primers. Therefore, ISSR-PCR was performed at an initial denaturation temperature of 94 °C for 5 min, 38 cycles of 94 °C for 30 s, 48–58 °C (depending on primer sequence) for 40 s and 72 °C for 1 min and a final extension of 72 °C for 10 min.

In the present study, SSRs developed in *S. austrocaledonicum* (Bottin et al. 2005) and *S. Insulare* (Emeline et al. 2006) were exploited for diversity analysis in sandalwood. PCRs for SSR were carried out in a final of volume of 10 μl containing 20 ng template DNA, 1× PCR buffer, 0.2 mMdNTPs, 0.5 pM of each primer, and 0.1 U *Taq polymerase* (Dream Taq, Thermo Scientific, USA). PCR was carried out with following programming: 94 °C for 3 min (denaturation), followed by 35 cycles of 94 °C for 30 s, 48–58 °C for 30 s, 72 °C for 1 min, and a final extension at 72 °C for 5 min.

Amplified products were electrophoresed in 1.5 % agarose for (RAPD and ISSR) and 2.5 % for SSR in 1× TBE buffer. The gels were stained with ethidium bromide and documented using gel documentation system (Bio-Rad, Hercules, California). Each experiment was repeated two times with each primer and those primers which gave reproducible fingerprints (DNA bands) were only considered for further experimentation and data analysis.

### Data analysis

For each genotype, each fragment/band that was amplified using primers was treated as unit character. Unequivocally reproducible bands were scored and entered into a binary character matrix (1 for presence and 0 for absence). The pairwise genetic similarity coefficient (GS) was calculated using Jaccard coefficient (Jaccard 1908) by the SIMQUAL program of NTSYS-pc software version 2.02 (Rohlf 1998). A dendrogram was constructed based on the matrix of distance using Unweighted Pair Group Method with Arithmetic averages (UPGMA).

To compare the efficiency of primers, polymorphic information content (PIC), as a marker discrimination power, was computed using the formula PIC = 1−∑*P*_*i*_^2^, where pi is the frequency of the *i*th allele at a given locus (Anderson et al. 1993). The PIC values are commonly used in genetics as a measure of polymorphism for a marker locus using linkage analysis. Correlation between the matrices obtained by marker types was estimated by means of Mantel test using MxComp module of NTSYSpc.

## Results and discussion

Forest and trees are renewable resources and contribute substantially to economic development. Overexploitation of forests for commercial purposes and other developmental activities have resulted in serious threat to tree species including sandal wood. In four decades, sandalwood production slumped from 4000 to 400 tonne a year (Times of India 2012). Overexploitation and poaching pushed this commercial forest tree in vulnerable category of the IUCN Red List (Kumar et al. 2012). Prohibition on export caused smuggling of sandalwood. The government of India’s Godowns have 15,000 tonnes of seized stock of sandalwood worth 5000 crore Indian rupees value in 2013 (Mahammadh 2014). Sandalwood grows naturally in the forest and since there is no systematic cultivation, this tree is at the face of increased exploitation. Therefore, genetic diversity analysis is essential for both the long-term stability and short-term productivity of trees as diversity provides clues to the factors that direct the variation, inbreeding and gene flow. The efforts to conserve decreasing genetic resources suffer from lack of precise information on genetic diversity (Naseer et al. 2012).

### RAPD-based diversity analysis

The data collected from 25 RAPD primers produced 225 total bands, of which 181 (83.13 %) were polymorphic (Table 2). Dani et al. (2011) obtained only 65.99 % polymorphism, which indicated that presently studied genotypes are more diverse. High polymorphic bands have been observed in many woody tree species with similar life cycles (Lacerda et al. 2001; Shrestha et al. 2002). However, the polymorphism level was low than stated by Suma and Balasundaran (2004) where 91.67 % of the RAPD loci were polymorphic. Previously, it has been reported that genetic diversity was higher among states of South India. The molecular size of the amplified PCR products ranged from 109 (OPP 14) to 2251 bp (OPF 05). Average numbers of loci per primer were nine and average numbers of polymorphic loci obtained per primer were found to be 7.12. The PIC values ranged from 0.70 (OPP 04) to 0.92 (OPA 02) with an average of 0.84. Primer OPA 02 generated maximum 15 loci.

**Table 2:** RAPD-based primers, total bands, polymorphic bands and PIC values

Primer name	Primer sequence (5′–3′)	Amplicon size (bp)	Total number of bands	Number of polymorphic bands	Polymorphism (%)	PIC value
OPA-02	TGCCGAGCTG	159–1387	15	8	53.33	0.92
OPA-03	AGTCAGCCAC′	210–1155	6	5	83.33	0.79
OPA-05	AGGGGTCTTG	220–2019	10	10	100	0.88
OPA-07	GACGGATCAG	190–1604	7	7	100	0.81
OPA-15	TTCCGAACCC	220–1228	7	7	100	0.84
OPC-03	GGGGGTCTTT	196–1575	9	5	83.33	0.89
OPC-07	GTCCCGACGA	139–2192	6	5	83.33	0.81
OPC-10	TGTCTGGGTG	240–1250	8	8	100	0.87
OPC-16	CACACTCCAG	190–838	7	6	85.71	0.84
OPD-02	GGACCCAACC	173–1993	9	9	100	0.89
OPD-03	GTCGCCGTCA	215–1295	11	11	100	0.89
OPD-05	ACCAGGTTGG	226–1325	7	6	85.71	0.86
OPD-08	GTGTGCCCCA	219–1561	7	7	100	0.8
OPD-18	GAGAGCCAAC	184–1398	8	5	62.5	0.86
OPD-20	ACCCGGTCAC	383–1208	7	5	71.42	0.83
OPE-03	CCAGATGCAC	641–1022	7	4	57.14	0.82
OPE-06	GGGTAACGCC	302–2017	8	7	87.5	0.81
OPE-15	ACAACGCCTC	229–1367	12	5	55.55	0.88
OPF-05	CCGAATTCCC	211–2251	12	10	83.33	0.9
OPF-08	GGGATATCGG	122–2236	14	13	92.86	0.89
OPP-04	GTGTCTCAGG	206–892	7	5	71.42	0.7
OPP-06	GTGGGCTGAC	169–1133	10	9	90	0.85
OPP-08	ACATCGCCCA	236–1029	10	6	60	0.88
OPP-14	CCAGCCGAAC	109–442	11	9	81.82	0.83
OPM-02	ACGCACAACC	206–1231	10	9	90	0.8
		Total	225	181	–	–
		Average	9	7.12	83.13	0.84

**Fig. 2:**
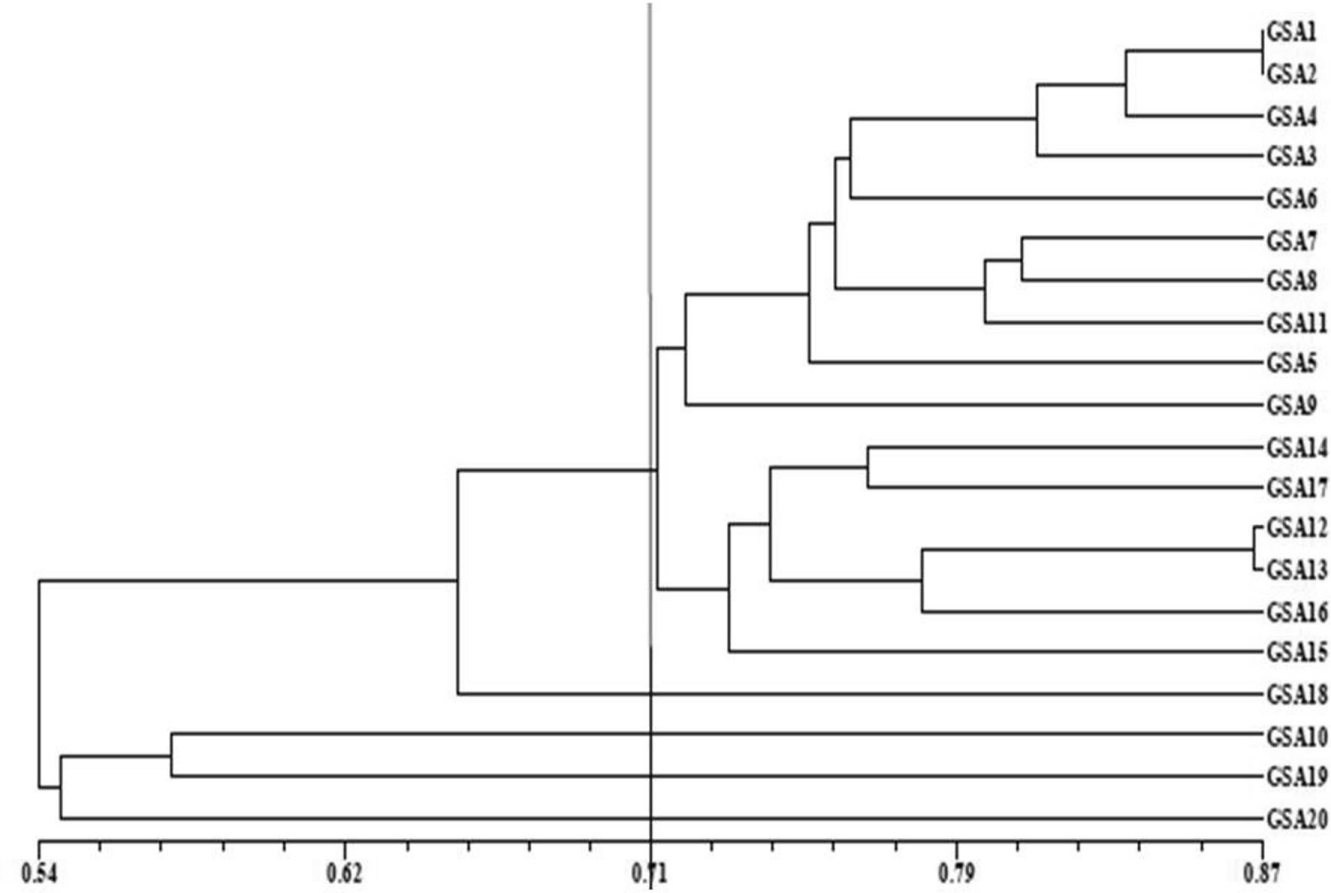
RAPD-based dendrogram of the genetic similarities among twenty accessions of sandalwood achieved by the UPGMA method (*Vertical line* cluster difference cutoff value of 0.71)

Jaccard’s similarity coefficients based on RAPD markers among the all pairwise combinations of genotypes ranged from 0.42 (GSA10/GSA15) to 0.87 (GSA1/GSA2) with a mean of 0.79 (Table 3). The results are in agreement with Suma and Balasundaran (2004) where relative magnitude of genetic similarity within populations was 0.77. The UPGMA clustering algorithm based on RAPD data grouped 20 genotypes into five clusters at cutoff value of 0.71 (Fig. 2). The RAPD-based dendrogram showed that cluster I consisted of maximum 16 genotypes of sandalwood and genotypes GSA 1 and GSA 2 (from Anand region) and GSA12 and GSA13 (from Vadodara region) clustered together. However, clusters II, III, IV and V each comprised of only one genotype. The results obtained in the present investigation are almost in agreement with the results of Azeez et al. (2009) and Dani et al. (2011).

**Table 3:** Similarity matrix of 20 sandalwood genotypes based on ISSR markers (upper diagonal) and RAPD markers (lower diagonal)

Genotype	GSA 1	GSA 2	GSA 3	GSA 4	GSA 5	GSA 6	GSA 7	GSA 8	GSA 9	GSA 10	GSA 11	GSA 12	GSA 13	GSA 14	GSA 15	GSA 16	GSA 17	GSA 18	GSA 19	GSA 20
GSA 1	1	0.753	0.721	0.716	0.701	0.69	0.645	0.569	0.636	0.604	0.662	0.74	0.648	0.638	0.634	0.631	0.639	0.605	0.571	0.636
GSA 2	0.872	1	0.701	0.749	0.694	0.636	0.683	0.639	0.665	0.643	0.689	0.667	0.725	0.636	0.663	0.688	0.743	0.663	0.604	0.665
GSA 3	0.807	0.804	1	0.752	0.785	0.68	0.712	0.674	0.692	0.656	0.685	0.741	0.737	0.784	0.738	0.652	0.69	0.624	0.6	0.625
GSA 4	0.874	0.854	0.823	1	0.72	0.677	0.742	0.609	0.667	0.614	0.66	0.669	0.698	0.691	0.697	0.681	0.718	0.644	0.632	0.615
GSA 5	0.736	0.764	0.738	0.771	1	0.762	0.715	0.711	0.708	0.704	0.745	0.72	0.705	0.687	0.764	0.667	0.673	0.64	0.607	0.684
GSA 6	0.748	0.759	0.744	0.796	0.756	1	0.762	0.656	0.63	0.671	0.656	0.686	0.663	0.642	0.649	0.596	0.614	0.64	0.576	0.62
GSA 7	0.775	0.746	0.73	0.753	0.786	0.748	1	0.664	0.694	0.691	0.665	0.641	0.693	0.685	0.68	0.665	0.692	0.669	0.603	0.576
GSA 8	0.769	0.777	0.769	0.755	0.763	0.768	0.807	1	0.69	0.676	0.649	0.604	0.635	0.657	0.653	0.618	0.646	0.622	0.608	0.591
GSA 9	0.701	0.699	0.777	0.726	0.705	0.683	0.756	0.757	1	0.638	0.644	0.688	0.662	0.688	0.637	0.634	0.662	0.617	0.648	0.607
GSA 10	0.495	0.52	0.497	0.523	0.553	0.483	0.503	0.556	0.684	1	0.654	0.609	0.65	0.596	0.636	0.613	0.64	0.616	0.603	0.671
GSA 11	0.78	0.867	0.724	0.765	0.755	0.741	0.797	0.798	0.71	0.497	1	0.775	0.699	0.681	0.81	0.715	0.688	0.654	0.599	0.678
GSA 12	0.73	0.758	0.694	0.765	0.735	0.796	0.759	0.737	0.699	0.595	0.758	1	0.743	0.739	0.732	0.68	0.675	0.652	0.617	0.676
GSA 13	0.734	0.75	0.704	0.737	0.746	0.694	0.768	0.749	0.69	0.573	0.771	0.87	1	0.771	0.728	0.721	0.758	0.737	0.682	0.718
GSA 14	0.759	0.697	0.659	0.727	0.75	0.689	0.672	0.694	0.637	0.538	0.725	0.754	0.746	1	0.761	0.658	0.675	0.697	0.686	0.676
GSA 15	0.775	0.774	0.657	0.696	0.726	0.782	0.675	0.685	0.62	0.425	0.767	0.706	0.737	0.731	1	0.721	0.714	0.691	0.657	0.706
GSA 16	0.73	0.73	0.684	0.699	0.706	0.796	0.726	0.727	0.67	0.581	0.739	0.779	0.781	0.743	0.766	1	0.821	0.665	0.621	0.645
GSA 17	0.72	0.699	0.663	0.698	0.705	0.656	0.705	0.688	0.65	0.503	0.71	0.749	0.793	0.765	0.695	0.749	1	0.779	0.66	0.673
GSA 18	0.665	0.658	0.655	0.689	0.64	0.657	0.659	0.661	0.603	0.497	0.637	0.654	0.624	0.637	0.64	0.673	0.722	1	0.752	0.66
GSA 19	0.554	0.535	0.55	0.547	0.531	0.533	0.622	0.567	0.556	0.577	0.539	0.636	0.606	0.553	0.538	0.668	0.604	0.634	1	0.707
GSA 20	0.536	0.526	0.551	0.52	0.53	0.48	0.547	0.549	0.548	0.531	0.521	0.497	0.531	0.554	0.491	0.574	0.538	0.523	0.565	1

High variation in sandalwood is usually related with geographic occurrence, habitat fragmentation and vegetative reproduction (Dani et al. 2011). The genetic diversity detected in the present study could be due to distance factor as the genotypes studied were widely distributed in different regions. Moreover, the heterozygous and heterogeneous structure of sandalwood population driven by its out breeding behaviour might be reason for high degree of polymorphism variability (Shashidhara et al. 2003).

### ISSR profile and diversity analysis

The data collected from ISSR markers with 21 arbitrary primers produced 208 total loci, of which 156 (75.77 %) were polymorphic (Table 4). Average numbers of loci per primer were found to be 9.90 and average numbers of polymorphic loci obtained per primer were found to be 7.42. The molecular size of the amplified PCR products ranged from 66 (ISD 21) to 1980 bp (UBC 815). The PIC values ranged from 0.79 (ISD 7 and UBC 890) to 0.91 (ISD 4, UBC 811, UBC 818, and UBC 834) with an average of 0.86. The values of observed PIC were in congruence with PIC (0.76–0.95) in 30 *Jatropha* accessions (Tanya et al. 2011). Marker UBC 858 generated maximum 14 loci.

**Table 4:** ISSR-based primers, total bands, polymorphic bands and PIC values

Primer name	Primer sequence (5′–3′)	Amplicon size (bp)	Total number of bands	Number of polymorphic bands	Polymorphism (%)	PIC value
ISD-1	GAGAGAGAGAGAGG	186–1485	10	8	80	0.88
ISD-3	GAGAGAGAGAGACC	128–1626	11	6	55	0.89
ISD-4	GTGTGTGTGTGTCC	79–1133	12	9	75	0.91
ISD-7	CTCTCTCTCTCTCTCTAC	113–879	8	5	63	0.79
ISD-16	AGAGAGAGAGAGAGAGC	378–1890	11	11	100	0.9
ISD-21	ACACACACACACACACTG	66–1232	6	5	83	0.81
ISD-36	GATAATACGAGAGAGAGAGAGA	92–1534	9	8	89	0.82
ISD-50	GACGACGACGACG	843–1052	10	7	70	0.89
UBC-808	AGAGAGAGAGAGAGAC	73–1364	10	8	80	0.84
UBC-811	GAGAGAGAGAGAGAGAC	127–1345	13	9	69	0.91
UBC-813	ACACACACACACACACT	132–1759	12	8	67	0.88
UBC-814	CTCTCTCTCTCTCTA	75–542	7	3	43	0.82
UBC-815	CTCTCTCTCTCTCTCTG	277–1980	7	7	100	0.8
UBC-816	CACACACACACACACAT	106–1796	13	12	92	0.89
UBC-818	CACACACACACACACAG	114–1349	12	5	42	0.91
UBC-822	TCTCTCTCTCTCTCTCA	153–1006	10	7	70	0.86
UBC-825	ACACACACACACACACT	127–609	7	6	86	0.83
UBC-834	AGAGAGAGAGAGAGAGAT	97–1433	12	6	50	0.91
UBC-858	TGTGTGTGTGTGTGTGRT	108–1831	14	13	93	0.9
UBC-888	BDBCACACACACACACA	128–694	7	6	86	0.85
UBC-890	VHVGTGTGTGTGTGTGT	188–1433	7	7	100	0.79
		Total	208	156	–	–
		Average	9.9	7.42	75.77	0.86

**Fig. 3:**
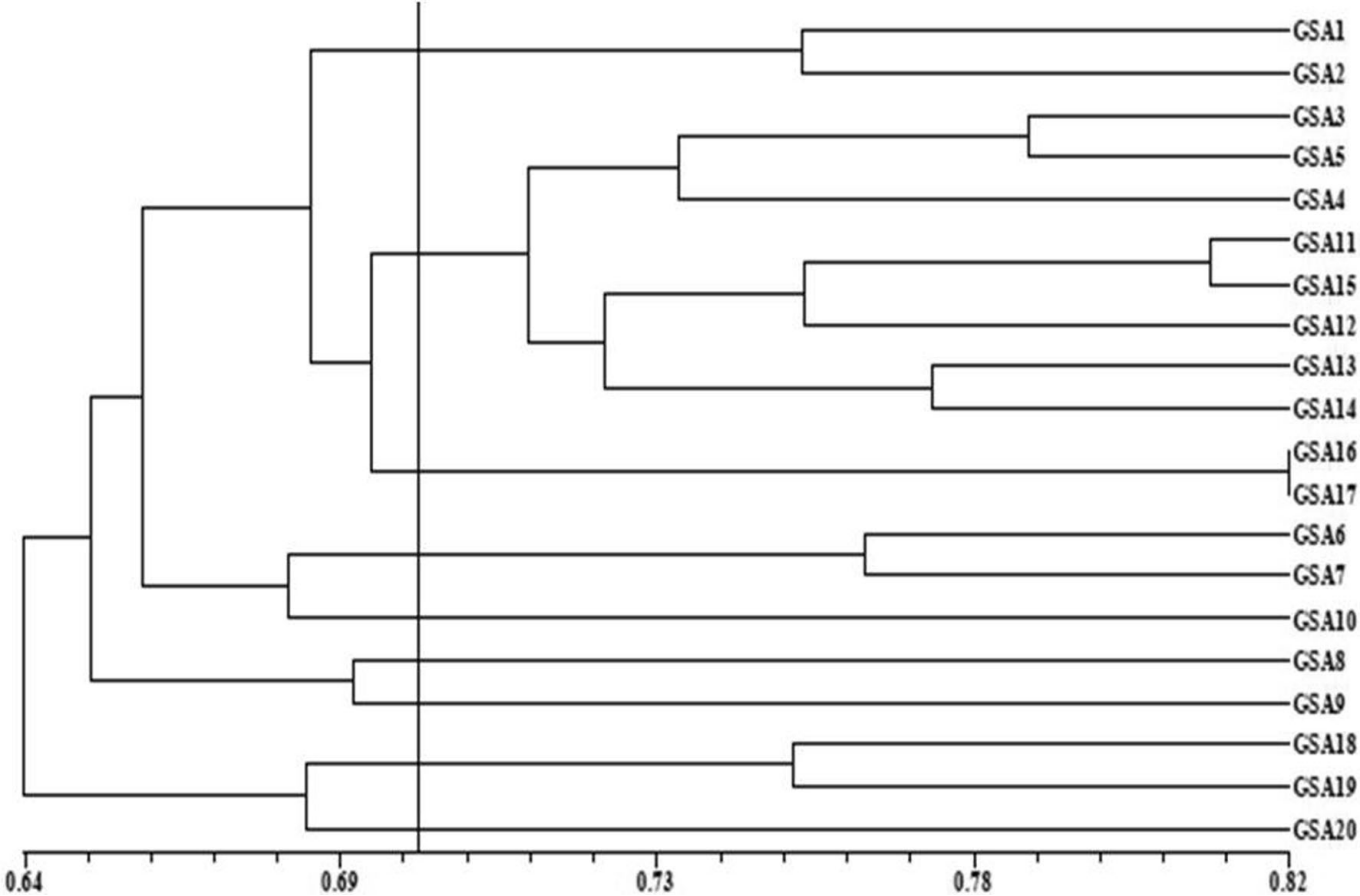
ISSR-based dendrogram of the genetic similarities among twenty accessions of sandalwood achieved by the UPGMA method (*Vertical line* cluster difference cutoff value of 0.70)

The similarity coefficient value ranged from 0.57 (GSA 1 and GSA19) to 0.81 (GSA 11 and GSA 15) indicating that the distribution of variation was diverse (Table 3). The average coefficient similarity for all the genotypes was found to be 0.70 (Fig. 3). Arif et al. (2009) obtained 0.56 to 0.93 similarity coefficient value in tree *Dualbergia sissoo*. The ISSR-based UPGMA clustering algorithm grouped genotypes in nine clusters at a cutoff value of 0.70. Maximum eight genotypes were grouped into cluster II. Cluster III harboured two genotypes (GSA 16 and GSA 17) at 100 % similarity coefficient. The results obtained in the present study portray slightly less polymorphism level compared to Basha and Sujatha (2007) but higher than Tanya et al. (2011), Gautan et al. (2013) in Jatropha.

### SSR analysis

During cross-species amplification in sandalwood, out of 16 SSR of *S. austrocaledonicum* and *S. insulare,* 11 primers gave successful amplification thus revealing transferability of SSR markers (Table 5). The cross species transferability results indicated that the sequences flanking the microsatellite regions in *Santalum* are highly conserved across species. The success rate is in agreement with Naseer et al. (2012) where out of 16, 12 primers amplified SSR loci in 20 genotypes of *S. album*. Further, examination of the polymorphism for the microsatellite primer pairs within sandalwood showed lack of genetic variation indicating the highly conserved nature of these loci in genus *Santalum*. Naseer et al. (2012) also found single and monomorphic locus for six SSRs. Similar results have been also reported in tree species by many researchers. While assessing cross-species amplification of SSRs from eucalyptus to *Casuarina equisetifolia*, Yasodha et al. (2005) found monomorphism for all the locus-specific products. Similarly, no genetic variation was observed by Rao et al. (2007b) during cross-species amplification of coconut SSRs in rattans. Recently, efforts were made to elucidate information from monomorphic markers (Holla et al. 2014) through nucleotide variations in monomorphic amplicons of SSR. Thus, monomorphic markers which are usually eliminated from the further study could be the ones that are associated with the trait of interest. In addition, the monomorphic primers need to be tested in a larger set of isolates.

**Table 5:** Result of amplified cross-species-transferred SSR primers with their sequences and amplicon size in sandalwood (*S. album*)

Primer name	Primer sequence (Forward**/**Reverse; 5′–3′)	Annealing temperature	Product size in native species	Product size on *S. album*
mSaCIRG01	GCTCAACCCATTTTTATCC**/**ACACAGCAGAACTCCAACA	52.4/54.5	273	288
mSaCIRG10	GTGCTACCTGCTACCCTTTTT**/**CCAATAACGGCTTCAACTTCA	57.9/55.9	247	240
mSaCIRF04	TCATTACACAGGCATCAGAAA**/**CTACCATCCACCACCGACAT	54/56	229	202
mSaCIRF10	TTAGGAAAACATAGCACACT**/**GAGCACTTCACCACCATTAC	51.2/57.0	155	153
mSaCIRH10	AAGCCCGATAACGAGAAAAGA**/**ATGAATAGGGATGGCGAGAGGT	57.1/60.6	219	242
mSiCIR33	GAAGTTGAAGTTGTTGATGC**/**AAATGAGAGACCTGAGTGAAG	53.2/55.9	220	212
mSaCIRH09	GCCTCTGCTTCCTCCCATTGTAG**/**AACTCCATTTGTGATTCCTCCCA	64.2/58.9	109	121
mSaCIRE09	GGAAAGGGTTGACAGGAAGAA**/**TGCGAGTGAGTGGGAAAAGTA	58.9/58.9	170	169
mSiCIR185	ACAACAACGCATAACCCT**/**AAAACAATGGCACTGAGAA	50.2/50.2	282	282
mSiCIR148	CATAGAAGTAGTTGGGTTTA**/**TTTTAGGTAGGATGTTGG	49.1/49.1	186	188
mSiCIR139	GTGCTACTTGATACCCAGG**/**GGACAACCAGAGGAGAAC	56.7/57.0	200	198

### Correlation between RAPD and ISSR markers and pooled clustering analysis

Similarity was observed to be high among genotypes on the basis of RAPD (0.79) and ISSRs (0.70) with average genetic variation up to 21 and 30 %, respectively. This was also reflected by high correlation (*r* = 0.96) between RAPD and ISSR analysis. High correlation values between two marker systems have been reported earlier in many plants species (Abdelhamid et al. 2014). High correlation might be due to the fact that both molecular types are dominant markers and that each marker system samples a very small fraction of the genome that was arbitrarily amplified (Table 6).

**Table 6:** Similarity matrix of 20 sandalwood genotypes based on pooled ISSR and RAPD marker analysis

Genotype	GSA 1	GSA 2	GSA 3	GSA 4	GSA 5	GSA 6	GSA 7	GSA 8	GSA 9	GSA 10	GSA 11	GSA 12	GSA 13	GSA 14	GSA 15	GSA 16	GSA 17	GSA 18	GSA 19	GSA 20
GSA 1	1																			
GSA 2	0.814	1																		
GSA 3	0.767	0.754	1																	
GSA 4	0.770	0.803	0.789	1																
GSA 5	0.720	0.730	0.759	0.747	1															
GSA 6	0.718	0.700	0.714	0.740	0.758	1														
GSA 7	0.684	0.716	0.722	0.748	0.713	0.754	1													
GSA 8	0.673	0.710	0.721	0.685	0.739	0.716	0.740	1												
GSA 9	0.671	0.679	0.703	0.699	0.706	0.659	0.728	0.727	1											
GSA 10	0.547	0.581	0.573	0.567	0.625	0.571	0.592	0.591	0.625	1										
GSA 11	0.725	0.755	0.706	0.716	0.750	0.702	0.735	0.728	0.680	0.571	1									
GSA 12	0.735	0.714	0.715	0.720	0.728	0.700	0.681	0.675	0.694	0.602	0.766	1								
GSA 13	0.692	0.738	0.719	0.719	0.726	0.679	0.706	0.694	0.677	0.611	0.737	0.809	1							
GSA 14	0.680	0.669	0.713	0.703	0.721	0.668	0.678	0.677	0.659	0.566	0.705	0.748	0.757	1						
GSA 15	0.679	0.691	0.690	0.696	0.741	0.687	0.677	0.671	0.623	0.523	0.757	0.718	0.733	0.744	1					
GSA 16	0.683	0.710	0.669	0.691	0.687	0.657	0.697	0.676	0.654	0.564	0.728	0.732	0.752	0.688	0.745	1				
GSA 17	0.680	0.716	0.676	0.707	0.689	0.636	0.699	0.668	0.656	0.572	0.699	0.713	0.738	0.722	0.704	0.783	1			
GSA 18	0.636	0.660	0.640	0.664	0.640	0.649	0.664	0.642	0.610	0.557	0.645	0.652	0.677	0.665	0.664	0.669	0.750	1		
GSA 19	0.562	0.568	0.574	0.586	0.566	0.553	0.608	0.586	0.598	0.590	0.566	0.627	0.642	0.612	0.592	0.619	0.631	0.690	1	
GSA 20	0.584	0.594	0.587	0.566	0.604	0.546	0.561	0.570	0.576	0.606	0.594	0.580	0.621	0.611	0.590	0.608	0.605	0.592	0.634	1

**Fig. 4:**
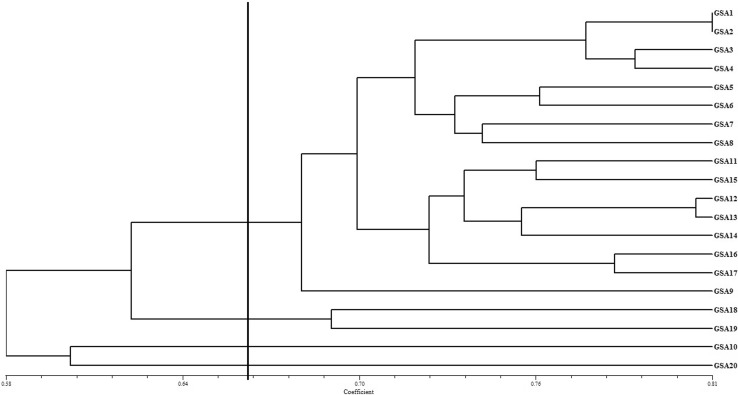
Pooled (RAPD and ISSR data based) dendrogram of the genetic similarities among twenty accessions of sandalwood achieved by the UPGMA method (*Vertical line* cluster difference cutoff value of 0.66)

Due to high correlation, UPGMA cluster analysis was also carried out using pooled RAPD and ISSR data. Jaccard’s similarity coefficient in pooled analysis ranged from 0.52 to 0.81 with a mean of 0.67. During pooled RAPD and ISSR analysis, Desai et al. (2015) also observed similar similarity coefficient range in bamboo. The UPGMA clustering algorithm based on pooled data grouped 20 genotypes into three clusters at cutoff value of 0.66 (Fig. 4). Clustering pattern derived using both the markers were found more or less similar when compared to the pooled RAPD and ISSR dendrogram. Desai et al. (2015) have also observed similar results in bamboo.

## Conclusion

The present study using RAPD and ISSR presented some valuable information about sandalwood diversity. Cross-species transferability of SSR indicated the conservation of primer binding site across the genera. However, compared to less reproducible marker, there is necessity to develop suitable and highly reproducible genomic resources viz. SSR or SNP in *S. album* for better genome coverage and for unravelling its variability to understand species relationships and for germplasm conservation.
